# Predictive Factors of Functioning Adrenal Incidentaloma: A 15-Year Retrospective Study

**DOI:** 10.3390/medicina58050597

**Published:** 2022-04-27

**Authors:** Natwara Muangnoo, Worapaka Manosroi, Napitch Leelathanapipat, Tanaporn Meejun, Pattanan Chowchaiyaporn, Pasinee Teetipsatit

**Affiliations:** 1 Department of Internal Medicine, Faculty of Medicine, Chiang Mai University, Chiang Mai 50200, Thailand; fahnatwara1412@gmail.com (N.M.); napitch2000@gmail.com (N.L.); pantanaporn.me@gmail.com (T.M.); benz-pattanan@outlook.com (P.C.); minnie31343@hotmail.com (P.T.); 2 Endocrine Unit, Internal Medicine Department, Faculty of Medicine, Chiang Mai University, Chiang Mai 50200, Thailand

**Keywords:** adrenal incidentaloma, primary aldosteronism, Cushing syndrome, pheochromocytoma

## Abstract

*Background and Objectives*: Adrenal incidentaloma is an adrenal mass that is found incidentally in abdominal imaging studies. It is important to categorize whether the mass is a functioning or non-functioning incidentaloma to determine the appropriate management and follow-up. Our study aimed to identify predictive factors for functioning adrenal incidentaloma that could assist in early detection and in determining which patients may require hormonal investigations. *Materials and methods*: This 15-year retrospective study was performed in a tertiary care medical center. A total of 244 patients with adrenal incidentaloma were identified, of whom 88 had functioning adrenal incidentaloma. The patients’ clinical biochemical factors and radiographic parameters were reviewed. Multivariable analysis using logistic regression with backward stepwise selection analysis was performed. Results are presented as odds ratios (ORs) with 95% confidence interval (CI). Statistical significance was set at *p* < 0.05. *Results*: A significant clinical predictor for functioning adrenal incidentaloma is a history of hypertension (OR 2.72, 95% CI(1.53, 4.82)), while significant radiological predictors include mass size >4 cm (OR 2.20, 95% CI(1.20, 4.02)) and Hounsfield units (HU) < 10 (OR 2.47, 95% CI(1.23, 4.94)). *Conclusions:* These easy-to-obtain clinical and radiological predictors can be used to help identify functioning adrenal incidentaloma. In clinical practice, taking these factors into consideration could potentially reduce the number of investigations required to diagnose functioning adrenal incidentaloma.

## 1. Introduction

Adrenal incidentaloma (AI) is an adrenal tumor greater than 1 cm which is often initially detected incidentally during abdominal imaging studies, e.g., magnetic resonance imaging (MRI) or computed tomography (CT) scans, that were not intended for investigating adrenal diseases. Based on evidence from autopsy studies, the prevalence of AI had been reported to be approximately 8% [1]. In patients with AI, it is imperative to determine if the AI is either malignant or functioning as both those conditions are potentially lethal and may require early intervention including possible surgical management. Approximately 10–15% of AI are functioning tumors [2]. Functioning AI can be classified into four subtypes: primary aldosteronism, autonomous cortisol secretion (ACS) (previously known as subclinical Cushing’s syndrome), pheochromocytoma and sex-steroid secreting AI [3]. Primary aldosteronism and ACS are the two most common functioning tumors, while sex-steroid secreting tumors are rarely found in AI [3]. Recent clinical practice guidelines recommend that every patient with AI should be investigated for catecholamine excess (pheochromocytoma) and glucocorticoid excess (ACS). Investigation for mineralocorticoid excess (primary aldosteronism) should be conducted only in patients with hypertension and/or hypokalemia [4]. 

Presently, exhaustive diagnostic tests are required to confirm the presence of biochemical functioning tumors in AI. In primary aldosteronism, hormonal evaluation for plasma aldosterone concentration (PAC) and plasma renin activity (PRA) are needed. Serum cortisol after a 1 mg dexamethasone suppression test and metanephrine levels either from serum or 24 h urine collection are used to screen for ACS and pheochromocytoma, respectively [4]. These biochemical investigations may not be available in some institutions, requiring some patients with AI to be referred to other medical centers. Identification of easy-to-assess clinical features or development of easy-to-perform investigations to help clinicians identify probable functioning AI could help reduce hormonal investigations and also lead to cost- and time-savings.

Previous studies have reported that larger size adenomas, lower DHEAS levels and lower serum adiponectin levels indicate a higher probability of ACS [5,6,7]. One small study reported that Hounsfield units (HU) was a predictor of ACS and pheochromocytoma in patients with AI [8]. However, those studies did not incorporate clinical factors as predictors of functioning AI. The present research focused on identifying clinical and investigational predictors for functioning AI to facilitate early detection and reduce unnecessary hormonal investigations. 

## 2. Materials and Methods

A 15-year retrospective cohort study to identify diagnostic prediction factors for functioning AI was performed using data obtained from electronic medical records of all patients who had specific ICD-10 (Supplementary appendix) related to adrenal diseases at Maharaj Nakorn Chiang Mai Hospital from January 2006 through November 2021. Only the patients who had been diagnosed with AI and had undergone or referred for hormonal work-up were included. The study was conducted under the Declaration of Helsinki and the protocol was approved by the Faculty of Medicine, Chiang Mai University, Ethical Committee (Ethical number: 7892/2564). The inclusion criteria were patients older than 15 years who had been diagnosed with AI and undergone hormonal investigations. The exclusion criteria were (1) patients whose lab results were not available or were not completed and (2) presented for work-up due to other reasons not related to incidentaloma, e.g., severe hypertension, unexplained hypokalemia, suspected of pheochromocytoma or Cushing’s syndrome. 

### 2.1. Data Collection 

All the data were retrospectively collected from electronic medical records. Data collected included demographic information, medical history, and signs and symptoms related to functioning AI. Only the results of the initial hormonal workups for functioning AI were retrieved. PAC and PRA performed using the enzyme-linked immunoassay method (ELISA) (DIASource Immunoassays^®^, Nivelles, Belgium) had intra-assay variability of 4.7–8.5% for PAC and 5.3–7.1% for PRA. Patient preparation prior to PAC and PRA measurement were based on 2016 Endocrine Society guidelines [9]. In brief, PAC and PRA were collected between 0700 h and 0900 h. The patients should have normal potassium level of >3.5 mEq/L. Those who were currently taking beta-blockers, ACE inhibitors or ARBs were asked to discontinue these medications at least 2 weeks prior to the test. Patients using diuretics and mineralocorticoid receptor blockers were requested to discontinue those medications at least 4 weeks prior to the screening test. Only slow-release verapamil, hydralazine and/or α-blockers for hypertension control were allowed to be continued. Samples for PAC and PRA were collected from patients in the upright position after they had been seated for 5–15 min. Patients with an upright ARR greater than 20 ng/dL per ng/mL·h who also had a PAC of more than 15 ng/mL and who also had suppressed PRA underwent the normal saline suppression test for confirmation of primary aldosteronism. The confirmation test was performed by infusion of 500 mL of 0.9% NSS over 4 h while in the supine position or seated position. PAC was measured after completion of the infusion; patients with PAC greater than 10 ng/mL while supine or greater than 6 ng/mL while seated were diagnosed as having primary aldosteronism. Measurement of serum cortisol after a 1 mg dexamethasone suppression test was conducted by electrochemiluminescence immunoassay (ECLIA) (Elecsys^®^ Cortisol II assay (Roche Diagnostics, Basel, Switzerland) with an intra-assay variability of less than 10%. Patients were instructed not to take medications containing glucocorticoid substances for 48 h prior to blood being drawn for the test. For 24 h urine metanephrine and normetanephrine, the ELISA method (DIASource Immunoassays^®^, Nivelles, Belgium) was employed and had an intra-assay variability of 9–12% for metanephrine and 9–13% for normetanephrine. The reference values for metanephrine and normetanephrine were 350 and 600 µg/day, respectively. Patients were instructed to discontinue potentially interfering medications, e.g., anti-depressants, antipsychotics and sympathomimetic agents, for 48 h prior to the 24 h urine collection. Additionally, creatinine in the 24 h urine sample was measured along with metanephrines as an indication of an adequate urine sample. Prior to August 2017, three specimens of 24 h urine vanillylmandelic acid (VMA) was used instead of 24 h urine metanephrines. Other biochemical data were collected within the 6 months prior to or following the diagnostic work up for functioning AI, but not during periods of acute illness. Radiological features of AI included HU on plain CT, absolute and relative washout percentage, plus size and site of the mass. If there were multiple nodules in the images, only the largest was recorded. 

### 2.2. Definitions

Primary aldosteronism was diagnosed when screening and confirmation tests for primary aldosteronism were positive based on the previously mentioned Endocrine Society guidelines [9]. Briefly, screening results of an aldosterone-renin ratio (ARR) > 20 (ng/dL)/(ng/mL/h), PAC levels >15 ng/dL and PRA < 1.0 ng/mL/h, and post-infusion saline loading test PAC levels >10 ng/dL were used to confirm primary aldosteronism. ACS was diagnosed when serum cortisol following a 1 mg dexamethasone suppression test was >1.8 µg/dL. Pheochromocytoma was diagnosed based on 24 h urine metanephrine or normetanephrine levels of more than twice the upper normal ranges. Functioning AI was defined as at least one hypersecretion of aldosterone, glucocorticoid or catecholamine according to the aforementioned criteria. Non-functioning AI was defined as no hypersecretion of any of those hormones. A history of hypertension was defined as patients who had been treated with any anti-hypertensive medications or who had a history of blood pressure >140/90 mmHg for at least 3 consecutive measurements. Overweight was defined as BMI > 23 kg/m^2^. 

### 2.3. Statistical Analysis

The data were analyzed using STATA program version 15.0. The statistically significance level was set as a two-tailed *p*-value < 0.05. For categorical variables, counts or percentages are reported; for normally distributed continuous variables, means and standard deviations (SD) are presented; for non-normally distributed continuous variables, median and interquartile ranges (IQR) are shown. For continuous data, the univariable analysis was conducted using the independent t-test for normally distributed variables and the Mann–Whitney U test for non-normally distributed variables. For categorical variables, analysis was done using the Fisher-exact test. Multivariable analyses of the predictors for functioning AI were performed using binomial logistic regression analysis and are reported as odds ratios (ORs) with a 95% confidence interval (CI). Predictors with significant *p*-values in the univariable analysis as well as those with clinical significance related to AI were incorporated in the multivariable model. The final model was developed using the stepwise backward regression technique. For variables with missing data >20%, multiple imputation regression was used. The calculated sample size was based on the rule of 10 in which we presumed that 3 predictive factors of functioning AI would be identified and that at least 10 events should be used for each factor. Based on that, at least 30 patients with functioning AI needed to be included. With a prevalence of functioning AI of approximately 15%, at least 200 patients with AI were required for this study. 

## 3. Results

A total of 244 patients with AI were identified from 13,614 patients. Study flow is shown in Supplementary appendix. The majority were female (*n* = 131, 53.7%). The mean age was 55.7 ± 13.5 years. Of the total, 88 patients (36.1%) had functioning AI, while 156 patients had non-functioning AI (63.9%). Among the patients with functioning AI, primary aldosteronism was the most common hypersecreting hormone (*n* = 37, 15.2%), The second most common was pheochromocytoma (*n* = 28, 11.5%) followed by ACS (*n* = 23, 9.4%). Among non-functioning AI, 6.4% (*n* = 10) were adrenocortical carcinoma. Hypertension was the most common underlying disease (47.9%), followed by dyslipidemia (30.7%). Diabetes mellitus was significantly more frequently observed in functioning AI than in non-functioning AI patients (*p* = 0.03). Of the signs and symptoms of AI, hypertensive status (34%) followed by overweight (25%) were the two most common presentations. Hypokalemia was more commonly recognized in functioning AI than in non-functioning AI (*p* = 0.03). (Table 1) Among biochemical investigation results, there were no statistically significant differences between the functioning and non-functioning AI groups. In terms of radiological features, plain HU was significantly lower in functioning AI than non-AI patients (*p* < 0.05). (Table 2)

According to the multivariable analysis (Table 3), the only statistically significant clinical predictive factor for AI is history of hypertension (OR = 2.72, *p* = 0.001), while the statistically significant biochemical predictive factors for adrenal incidentaloma are HU > 10 (OR = 2.47, *p* = 0.011) and mass size > 4 cm (OR = 2.20, *p* = 0.010). 

## 4. Discussion

The present study found that both clinical factors and radiological features can assist in the prediction of functioning AI. According to the European Society of Endocrinology Guideline for AI, all patients with AI should undergo hormonal work-up to exclude pheochromocytoma and ACS [10]. In patients with hypertension or unexplained hypokalemia, screening for primary aldosteronism should also conducted [10]. However, this guideline did not mention the size and HU on plain CT as the indications for hormonal work-up. According to this guideline, the size and HU on plain CT were mentioned in terms of malignancy risk. Thus, the results found in the present study may help guide clinicians to consider which patients need to be referred for hormonal work-up particularly in institutions where hormonal tests may not be available.

However, some of the findings of this study do not agree with findings of previous studies. A prior cross-sectional study reported that a plain HU of ≥18.5 on non-contrast CT scans was a significant radiological parameter that could help detect functioning AI [8], while our study determined that patients with HU < 10 on plain CT scans have a probability of the mass being functioning 2.47 times that of patients with HU ≥ 10. Similar to our study, a previous report on 77 patients stated that a greater mass size of AI was significantly related to functioning AI, particularly the ACS subtype [11]. That study, however, did not specify the cut-off nodule size associated with a significantly increased risk of a functioning AI. Another study which included a larger cohort stated that AIs with a mass >3 cm were significantly related to the occurrence of functioning AI [12]. Biochemical factors reported to be associated with a functioning AI especially in ACS include lower DHEAs levels (<40 µg/dL) and lower adiponectin levels (<13 ng/mL) [6,7]. However, there factors were not collected in the present study. 

Patients with a history of hypertension had a risk of a functioning AI 2.72 times higher than patients with no history of hypertension. One possible explanation is that in our cohort most of the functioning AIs had also been diagnosed with primary aldosteronism, the most common clinical presentation of which is hypertension or resistant hypertension [13]. One study reported that approximately 10–15% of hypertensive patients were also diagnosed with primary aldosteronism [14]. Analysis of our data revealed that primary aldosteronism was more commonly found in hypertensives (81.1%) than non-hypertensives (42.0%) with *p* < 0.005. 

Radiological findings have shown that low unenhanced HU (<10) indicates a higher fat percentage in the incidentaloma which can indicate lipid-rich adenoma or benign adenoma. If HU is >10, lipid poor benign adenoma, metastasis, pheochromocytoma or complicated (hemorrhagic adenoma) should be suspected [15]. A previous study reported that plain or unenhanced HU > 18.5 was correlated with functioning AI, ACS and pheochromocytoma. The most common hypersecreting hormone in functioning AI in that study was ACS followed by pheochromocytoma and primary aldosteronism. The report clarified this finding, stating that in ACS, lipid-rich cortisol-producing cells are transformed to lipid-poor cells due to the reduction in intracytoplasmic fat during the excess cortisol production [16]. This transformation to lipid-poor cells has been reported to cause an increase in plain HU [16]. A probable explanation why their HU outcome is different from ours is that the majority of the functioning AI in their study was from ACS and pheochromocytoma, while in our study it was from primary aldosteronism. The plain HU in primary aldosteronism in the present study was less than 10 HU (range −10 to 12 HU) which suggests lipid-rich benign adenoma. Primary aldosteronism with high HU (>10) may suggest lipid-poor adenoma or carcinoma, both of which are rare [17]. Differentiation between lipid-rich and lipid-poor adenoma can be made by absolute washout or relative percentage washout using either enhanced CT or chemical-shift MRI [18]. 

In categorizing mass size, we used a standard cut-off of 4 cm because that value is widely used in the evaluation of the malignancy of a mass, and hence to determine the appropriate management for AI patients. Most previous reports have stated that there is a correlation between functioning AI and larger mass size [11,12]. For example, Barzon et al. stated that masses of >3 cm are linked to functioning AI [12]. Our finding showed similar result. Another study reported that there was no significant correlation between mass size and hormonal function as masses of all sizes are able to produce hormones [19]. The clarification for our finding on mass size was that the larger the size of an AI, particularly those >4 cm, the higher the risk of pheochromocytoma and adrenocortical carcinoma. Pheochromocytoma was found to be the second most common hypersecreting tumor in our cohort which could explain tumor size larger than 4 cm. For adrenocortical carcinoma, a rare type of adrenal mass, the most common hormonal hypersecretions are steroid precursors and cortisol, while some produce adrenal androgens [20]. 

A strength of this study is that the sample size was larger than most previously reported studies, providing greater power of analysis. Additionally, we combined both an easy-to-assess clinical indicators and easy-to-perform radiological evaluation as predictive factors for functioning AI which are widely available in other institutions. The results of this study can be employed in further research to develop a risk scoring system that incorporates both the clinical predictor and the radiological features identified in this study. Moreover, the significant relationship among the predictors can be explained by supportive evidence discussed previously. 

We acknowledge some limitations in this study. First, this was a retrospective study, and therefore may have introduced some biases. Second, it was a single-center study which limits the generalizability and applicability of the results to other institutions. Third, a diverse population of functioning AI patients were included which may have made the data less homogeneous and could potentially have resulted in lower predictive accuracy, e.g., a history of hypertension may predict only hyperfunctioning of aldosterone and catecholamines but not other functioning AI. On the other hand, that patient diversity suggests that the results would be applicable for diverse outpatient populations. Fourth, some radiological evaluation results were not available, e.g., contrast-enhanced HU as well as absolute and relative washout percentage. Fifth, the prevalence of functioning AI in this cohort is quite high. The presumption is during the registration of ICD-10 codes in patients with multiple diagnoses, the codes might not include all patients who were diagnosed with AI. 

## 5. Conclusions

A clinical history of hypertension as well as HU and adenoma size acquired from initial CT scans for AI can help predict the risk and aid early detection of a functioning AI mass. The process of obtaining these predictors is not difficult and can be carried out in most institutions. These predictors can provide guidance for general practitioners to consider in determining which patients needed to be referred for further hormonal investigations and which do not, resulting in time- and cost-savings. Further research is needed to develop a scale of clinical risk scores for patients with AI prior to using these predictive factors in a clinical setting.

## Figures and Tables

**Table 1:** Baseline demographic characteristics of patients with functioning adrenal incidentaloma (*n* = 88) and non-functioning adrenal incidentaloma (*n* = 156).

Characteristic	Functioning Adrenal Incidentaloma	Non-Functioning Adrenal Incidentaloma	* p *-Value *
Male, *n* (%)	42 (47.73)	71 (45.51)	0.74
Age (mean ± SD) (years)	55.09 ± 13.01	55.98 ± 13.87	0.62
BMI (mean ± SD) (kg/m^2^)	23.87 ± 5.38	22.78 ± 4.13	0.07
Heart rate (mean ± SD) (bpm)	85.66 ± 16.86	86.08 ± 15.70	0.84
SBP (mean ± SD) (mmHg)	131.85 ± 18.91	128.08 ± 20.49	0.16
DBP (mean ± SD) (mmHg)	78.41 ± 11.76	75.37 ± 12.45	0.06
Smoking status, *n* (%)	7 (7.95)	20 (12.82)	0.29
** Underlying diseases, *n* (%) **			
Hypertension	54 (61.36)	63 (40.38)	0.002
Coronary heart disease	6 (6.82)	5 (3.21)	0.21
Stroke	4 (4.55)	2 (1.28)	0.19
Heart failure	1 (1.14)	2 (1.28)	1.00
Diabetes mellitus	26 (29.55)	27 (17.31)	0.03
Dyslipidemia	32 (36.36)	43 (27.56)	0.19
Chronic kidney disease	7 (7.95)	6 (3.85)	0.23
** Signs and symptoms, *n* (%) **			
Atrial fibrillation	1 (1.14)	0 (0.00)	0.36
Sweating	2 (2.27)	2 (1.28)	0.62
Headache	4 (4.55)	1 (0.64)	0.05
Palpitation	4 (4.55)	5 (3.21)	0.72
Overweight	27 (30.68)	34 (21.79)	0.13
Hypertension	40 (45.45)	53 (33.97)	0.09
Cushingoid appearance	1 (1.14)	0 (0.00)	0.36
Muscle weakness	3 (3.41)	1 (0.64)	0.13
Documentation of hypokalemia	6 (6.82)	2 (1.28)	0.03
Atrial fibrillation	1 (1.14)	0 (0.00)	0.36

BMI: body mass index, SBP: systolic blood pressure, DBP: diastolic blood pressure, SD: standard deviation. * Univariate analysis.

**Table 2:** Baseline investigations of patients with functioning adrenal incidentaloma (*n* = 88) and non-functioning adrenal incidentaloma (*n* = 156).

Characteristic	Functioning Adrenal Incidentaloma	Non-Functioning Adrenal Incidentaloma	*p*-Value *
** Adrenal incidentaloma characteristics **			
** Site of adrenal incidentaloma, *n* (%) **			
Unilateral	78 (88.64)	140 (89.74)	0.83
Bilateral	10 (11.36)	16 (10.26)	
Adrenal incidentaloma size, median (IQR) (cm)	2.35 (1.4–7.3)	2.2 (1.3–4.45)	0.18
Plain Hounsfield unit, median (IQR) (unit)	29.84 (8–43.2)	39.00 (17.15–54.39)	0.0065
** Biochemical investigations **			
Fasting blood glucose, median (IQR) (mg/dL)	103 (91–128.34)	103 (91.87–119.13)	0.64
LDL, median (IQR) (mg/dL)	106.15 (88.85–125.94)	110.5 (85.63–130.92)	0.59
HDL, median (IQR) (mg/dL)	50.5 (39.58–60.66)	47.23 (36.80–58.20)	0.19
Triglyceride, median (IQR) (mg/dL)	141 (91.62–187.1)	125 (86.62–163.01)	0.18
Total cholesterol (mean ± SD) (mg/dL)	180.97 ± 41.08	176.51 ± 52.30	0.49
BUN, median (IQR) (mg/dL)	13 (9–17)	13 (9.5–16)	0.85
Creatinine, median (IQR) (mg/dL)	0.84 (0.72–1.08)	0.8 (0.7–1.03)	0.41
Hemoglobin (mean ± SD) (g/dL)	12.45 ± 1.95	12.37 ± 2.08	0.75
Albumin, median (IQR) (g/dL)	4.24 (4–4.46)	4.1 (3.74–4.41)	0.41
Serum potassium (mean ± SD) (mEq/L)	4.04 ± 0.51	4.08 ± 0.44	0.52
Serum sodium (mean ± SD) (mEq/L)	139.28 ± 3.87	138.62 ± 4.26	0.23
Serum bicarbonate (mean ± SD) (mEq/L)	24.91 ± 2.98	25.21 ± 3.03	0.47
** Incidentaloma functional investigation **			
Serum cortisol after 1 mg dexamethasone suppression test (mean ± SD) (µg/dL)	6.02 ± 7.37	1.65 ± 1.81	0.0001
Plasma aldosterone concentration (mean ± SD) (ng/dL)	183.99 ± 273.88	17.18 ± 22.2	0.0008
Plasma renin activity (mean ± SD) (ng/mL/h)	1.35 ± 4.18	1.55 ± 2.09	0.79
24 h urine metanephrine (mean ± SD) (µg/24-h)	742.39 ± 1972.31	154.66 ± 122.13	0.03
24 h urine normetanephrine (mean ± SD) (µg/24-h)	1706.06 ± 6621.56	329.07 ± 280.47	0.12
24 h urine VMA day 1 (mean ± SD) (mg/24-h)	19.55 ± 15.13	5.59 ± 3.65	0.0001
24 h urine VMA day 2 (mean ± SD) (mg/24-h)	13.98 ± 8.57	6.06 ± 4.67	0.0001
24 h urine VMA day 3 (mean ± SD) (mg/24-h)	17.47 ± 19.12	6.19 ± 4.69	0.0001

SD: standard deviation, VMA: vanillylmandelic acid. * Univariate analysis.

**Table 3:** Multivariable logistic regression analysis of predictive factors for adrenal incidentaloma.

Factor	OR	95% CI	* p *-Value
History of hypertension	2.72	1.53–4.82	0.001
Hounsfield units < 10	2.47	1.23–4.94	0.011
Size > 4 cm	2.20	1.20–4.02	0.010

OR: Odds ratio.

## Data Availability

The datasets generated and/or analyzed during the current study are available in https://www.dropbox.com/sh/93djfpf8667rvm8/AADVJb1lakHcoauAxKRtYYQpa?dl=0 (accessed on 1 March 2022).

## Supplementary Materials

The following supporting information can be downloaded at: https://www.mdpi.com/article/10.3390/medicina58050597/s1, Table S1: ICD-10 code.

## Abbreviations

ACS	Autonomous cortisol secretion
AI	Adrenal incidentaloma
CT	Computerized tomography
CI	Confidence interval
ECLIA	Electrochemiluminescence immunoassay
ELISA	Enzyme-linked immunoassay
HU	Hounsfield unit
MRI	Magnetic resonance imaging
OR	Odds ratio
SD	Standard deviation
VMA	Vanillylmandelic acid

## References

[1] Hedeland H., Ostberg G., Hökfelt B. (1968). On the prevalence of adrenocortical adenomas in an autopsy material in relation to hypertension and diabetes. Acta Med. Scand..

[2] Barzon L., Scaroni C., Sonino N., Fallo F., Gregianin M., Macrì C., Boscaro M. (1998). Incidentally discovered adrenal tumors: Endocrine and scintigraphic correlates. J. Clin. Endocrinol. Metab..

[3] Sherlock M., Scarsbrook A., Abbas A., Fraser S., Limumpornpetch P., Dineen R., Stewart P.M. (2020). Adrenal Incidentaloma. Endocr. Rev..

[4] Vaidya A., Hamrahian A., Bancos I., Fleseriu M., Ghayee H.K. (2019). The Evaluation of Incidentally Discovered Adrenal Masses. Endocr. Pract..

[5] Mintziori G., Georgiou T., Anagnostis P., Adamidou F., Efstathiadou Z., Panagiotou A., Kita M. (2018). Could Lipid Profile be Used as a Marker of Autonomous Cortisol Secretion in Patients with Adrenal Incidentalomas?. Horm. Metab. Res..

[6] Dogruk Unal A., Ayturk S., Aldemir D., Bascil Tutuncu N. (2016). Serum Adiponectin Level as a Predictor of Subclinical Cushing’s Syndrome in Patients with Adrenal Incidentaloma. Int. J. Endocrinol..

[7] Yener S., Yilmaz H., Demir T., Secil M., Comlekci A. (2015). DHEAS for the prediction of subclinical Cushing’s syndrome: Perplexing or advantageous?. Endocrine.

[8] Al-Waeli D.K., Mansour A.A., Haddad N.S. (2020). Reliability of adrenal computed tomography in predicting the functionality of adrenal incidentaloma. Niger. Postgrad. Med. J..

[9] Funder J.W., Carey R.M., Mantero F., Murad M.H., Reincke M., Shibata H., Stowasser M., Young W.F. (2016). The Management of Primary Aldosteronism: Case Detection, Diagnosis, and Treatment: An Endocrine Society Clinical Practice Guideline. J. Clin. Endocrinol. Metab..

[10] Fassnacht M., Arlt W., Bancos I., Dralle H., Newell-Price J., Sahdev A., Tabarin A., Terzolo M., Tsagarakis S., Dekkers O.M. (2016). Management of adrenal incidentalomas: European Society of Endocrinology Clinical Practice Guideline in collaboration with the European Network for the Study of Adrenal Tumors. Eur. J. Endocrinol..

[11] Bleier J., Pickovsky J., Apter S., Fishman B., Dotan Z., Tirosh A., Shlomai G. (2022). The association between adrenal adenoma size, autonomous cortisol secretion and metabolic derangements. Clin. Endocrinol..

[12] Barzon L., Scaroni C., Sonino N., Fallo F., Paoletta A., Boscaro M. (1999). Risk factors and long-term follow-up of adrenal incidentalomas. J. Clin. Endocrinol. Metab..

[13] Reincke M., Bancos I., Mulatero P., Scholl U.I., Stowasser M., Williams T.A. (2021). Diagnosis and treatment of primary aldosteronism. Lancet Diabetes Endocrinol..

[14] Calhoun D.A. (2006). Aldosteronism and Hypertension. Clin. J. Am. Soc. Nephrol..

[15] Francis I.R. (2003). Distinguishing benign from malignant adrenal masses. Cancer Imaging.

[16] Chambre C., McMurray E., Baudry C., Lataud M., Guignat L., Gaujoux S., Lahlou N., Guibourdenche J., Tissier F., Sibony M. (2015). The 10 Hounsfield units unenhanced computed tomography attenuation threshold does not apply to cortisol secreting adrenocortical adenomas. Eur. J. Endocrinol..

[17] Patel S.M., Lingam R.K., Beaconsfield T.I., Tran T.L., Brown B. (2007). Role of radiology in the management of primary aldosteronism. Radiographics.

[18] Seo J.M., Park B.K., Park S.Y., Kim C.K. (2014). Characterization of Lipid-Poor Adrenal Adenoma: Chemical-Shift MRI and Washout CT. Am. J. Roentgenol..

[19] Cyranska-Chyrek E., Szczepanek-Parulska E., Olejarz M., Ruchala M. (2019). Malignancy Risk and Hormonal Activity of Adrenal Incidentalomas in a Large Cohort of Patients from a Single Tertiary Reference Center. Int. J. Environ. Res. Public Health.

[20] Calissendorff J., Calissendorff F., Falhammar H. (2016). Adrenocortical cancer: Mortality, hormone secretion, proliferation and urine steroids—Experience from a single centre spanning three decades. BMC Endocr. Disord..

