# Supplementary material for: Predictive Factors of Functioning Adrenal Incidentaloma: A 15-Year Retrospective Study
Source: Medicina (Kaunas). 2022 Apr 27;58(5):597. doi: 10.3390/medicina58050597 (PMC9144654; doi:10.3390/medicina58050597)
Supplement: Supplementary file 1 [file medicina-58-00597-s001.zip › medicina-1706980-supplementary.pdf]

Supplementary appendix

Table S1 ICD-10 code

Disease	ICD code
Cushing’s syndrome,	(E24, E240, E248, E249)
Hyperaldosteronism	(E26, E260, E261, E268, E269),
Adrenal lesions	(C74, C740, C749, D350, D441, E351, E27, E270, E275, E278, E279),
Pheochromocytoma	(C741, C741, C749, D350, E275, R825),
Endocrine gland lesions	(C75, C759, D35, D357, D359).

Study flow

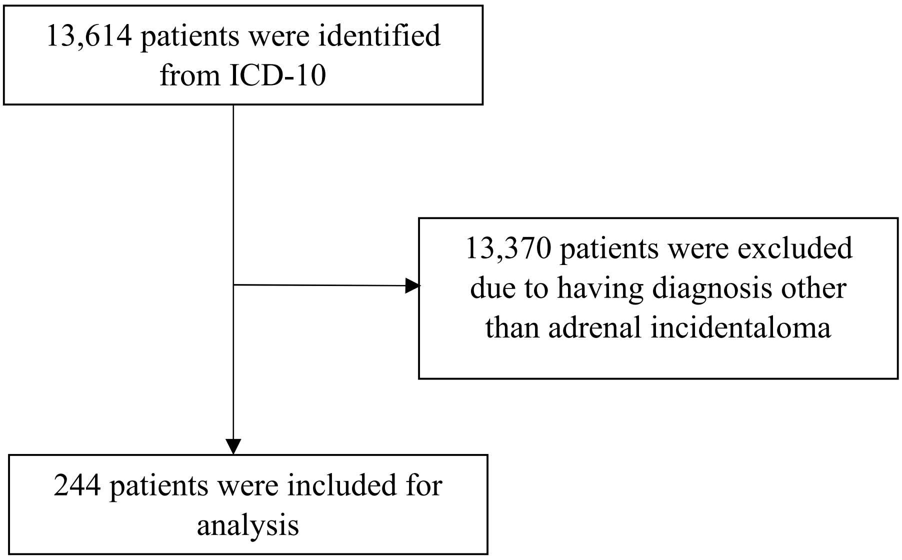
13,614 patients were identified from ICD-10
13,370 patients were excluded due to having diagnosis other than adrenal incidentaloma
244 patients were included for analysis
